# Predicting Prediabetes Through Facebook Postings: Protocol for a Mixed-Methods Study

**DOI:** 10.2196/10720

**Published:** 2018-12-14

**Authors:** Xiaomeng Xu, Michelle L Litchman, Perry M Gee, Webb Whatcott, Loni Chacon, John Holmes, Sankara Subramanian Srinivasan

**Affiliations:** 1 Department of Psychology Idaho State University Pocatello, ID United States; 2 College of Nursing University of Utah Salt Lake City, UT United States; 3 Nursing Research and Analytics Dignity Health San Francisco, CA United States; 4 College of Pharmacy University of Utah Salt Lake City, UT United States; 5 Department of Family Medicine Idaho State University Pocatello, ID United States; 6 David Eccles School of Business University of Utah Salt Lake City, UT United States

**Keywords:** diagnosis, Facebook, infodemiology, protocol, social media

## Abstract

**Background:**

The field of infodemiology uses health care trends found in public networks, such as social media, to track and quantify the spread of disease. Type 2 diabetes is on the rise worldwide, and social media may be useful in identifying prediabetes through behavior exhibited through social media platforms such as Facebook and thus in designing and administering early interventions and containing further progression of the disease.

**Objective:**

This pilot study is designed to investigate the social media behavior of individuals with prediabetes, before and after diagnosis. Pre- and postdiagnosis Facebook content (posts) of such individuals will be used to create a taxonomy of prediabetes indicators and to identify themes and factors associated with an actual diagnosis of prediabetes.

**Methods:**

This is a single-center exploratory retrospective study that examines 20 adults with prediabetes. The investigators will code Facebook posts 3 months before through 3 months after prediabetes diagnosis. Data will be analyzed using both qualitative content analysis methodology as well as quantitative methodology to characterize participants and compare their posts pre- and postdiagnosis.

**Results:**

The project was funded for 2015-2018, and enrollment will be completed by the end of 2018. Data coding is currently under way and the first results are expected to be submitted for publication in 2019. Results will include both quantitative and qualitative data about participants and the similarities and differences between coded social media posts.

**Conclusions:**

This pilot study is the first step in creating a taxonomy of social media indicators for prediabetes. Such a taxonomy would provide a tool for researchers and health care professionals to use social media postings for identifying those at greater risk of having prediabetes.

**International Registered Report Identifier (IRRID):**

DERR1-10.2196/10720

## Introduction

### Background

There are 86 million adults with prediabetes, with numbers rising in epidemic proportions. The Centers for Disease Control and Prevention has emphasized the need to confront prediabetes through National Diabetes Prevention Programs. In 2014, the Centers for Disease Control and Prevention reported that of the 37% of US adults who had prediabetes, 29% were unaware they had it and were therefore unlikely to seek out treatment [1]. When prediabetes is not diagnosed and treated accordingly, it can increase an adult’s risk of heart disease, stroke, and type 2 diabetes, all of which have their own economic and quality of life burdens. Enhanced epidemiologic approaches are needed to identify large populations who are unaware they have prediabetes to maximize the efficacy of diabetes prevention programs.

Around 7 in 10 American adults use social media [2]. Infodemiology, an epidemiologic approach that uses social media and other Web-based sources to examine the spread or incidence of disease [3,4], has been used to successfully predict important issues of public health such as depression and the likelihood of depression resulting in suicide among military service personnel [5-7], infectious diseases such as influenza [8,9], and the spread of interest in health issues such as Zika [10]. Using infodemiology for surveillance (ie, infoveillance) can be very effective in identifying many real-world health trends. However, this methodology has not yet been used to identify chronic conditions or precursors to chronic conditions, such as prediabetes. Infectious diseases often present with a sudden onset of symptoms while the development of chronic conditions is more subtle. Given the association between prediabetes and lifestyle and the fact that many individuals share their lifestyle on Facebook, this study uniquely examines the posts of those with prediabetes in the 3 months before and 3 months after their diagnosis. This innovative approach sets the stage to screen large populations for prediabetes who may have otherwise had a missed or delayed diagnosis.

### Prediabetes

Prediabetes is the stage between normal glucose levels and diabetes. There are a number of prediabetes risk factors noted in Textbox 1. Without the identification of prediabetes risk factors, confirmatory blood tests such as HbA_1c_, fasting plasma glucose, or a 2-hour glucose tolerance test [7] may not be ordered. Glucose levels can normalize if prediabetes is identified early and lifestyle changes are implemented. Therefore, timely diagnosis is imperative. Unfortunately, only 6% of primary care providers (PCPs) can accurately identify all prediabetes indicators [11], potentially resulting in missed or delayed diagnosis. Further, with the primary care shortage of 1 physician for every 2500 patients, lack of access to PCPs limits the capacity for the current health care system to make an appropriate and timely diagnosis [12]. Thus, a predictive model of prediabetes using social media postings will allow us to identify individuals in need of screening whom PCPs may have missed. Further, we will also be able to identify individuals who have not yet seen a health care provider but are at risk for prediabetes.

### Social Media

The majority of American adults use social media, and the social media digital divide is closing, as more adoption is increasing in baby boomers and older adults [2,13]. Further, social media use extends across ethnic and racial groups [2,14] due to mobile phone access.

Social media provides an outlet for individuals to share information about their lifestyle and health behaviors. This includes diet quality, activity, and sleep quality, all of which are associated with prediabetes [15-17]. For example, someone might post excessively about the movies and television shows they are watching, suggesting high levels of screen time and, thus, inactivity. While someone might post about the food they are eating, others might acknowledge unhealthy habits by sharing websites related to unhealthy eating. With the high rates of prediabetes and social media use, there is a high probability of a significant population of individuals with diagnosed and undiagnosed prediabetes using social media.

**Prediabetes risk factors.**

- Overweight, body mass index >25 kg/m^2^
- Age ≥45 years
- Hypertension
- Dyslipidemia
- Heart disease
- Family history of type 2 diabetes in a first-degree relative
- Sedentary lifestyle
- Smoking
- History of gestational diabetes or giving birth to a baby >9 pounds
- Polycystic ovarian syndrome
- African American or Asian race
- Hispanic ethnicity

This study will examine Facebook users with prediabetes and investigate their health behavior through the examination of Facebook posts 3 months before and 3 months after a diagnosis of prediabetes. Since this is a pilot study and the first infodemiology study to examine social media content and prediabetes, we decided to focus on 1 social media platform. We chose Facebook, as 8 in 10 adults use it [13] with substantial numbers across all age groups including 26.5 million users aged 55-64 years and 21.1 million users 65 years or older [18]. It is also a social media platform used worldwide, and, thus, health research and interventions using Facebook have the potential to influence large numbers of people.

### Proposed Research

The proposed study is rooted in populomics: the study of social interactions that either result in disease or protect health on a population level. This multidisciplinary field incorporates the study of population-level risk characterization (eg, prediabetes behaviors or indicators) through the use of information technology. This knowledge is then used to support public health interventions [19]. Using Facebook data, we will address the need for early diagnosis in prediabetes in order to prevent progression to diabetes.

We propose to address gaps in prediabetes infodemiology by examining indicators of prediabetes in the Facebook posts of individuals before and after a diagnosis of prediabetes. Our overall hypothesis is that individuals with prediabetes will have indicators of prediabetes on Facebook postings prior to diagnosis. Thus, the purpose of our study is 2-fold: (1) develop a taxonomy of prediabetes indicators and (2) explore if prediagnosis Facebook data among individuals already diagnosed with prediabetes can predict a trajectory toward prediabetes.

## Methods

### Design

This is an exploratory retrospective study that will examine Facebook posts among individuals with prediabetes. Comparisons of prediabetes indicators before and after prediabetes diagnosis will be analyzed.

### Participants

The setting is a family medicine clinic and community health center that is located in Southeastern Idaho, USA. The sample will be 20 adults with prediabetes. This sample size was chosen as this is an exploratory pilot study and the first infodemiology study to examine prediabetes and Facebook usage. Additionally, in this study, we aim to identify prediabetes indicators in social media data that can be verified with future large-scale studies. Furthermore, this pilot study will provide important information for future research on the feasibility of conducting infodemiology research on a chronic condition.

To be included, participants must be between the ages of 18 and 89 years, be able to read and write English, and be Facebook users who have been using Facebook for at least 3 months prior to their prediabetes diagnosis. Participants will have a medical record of prediabetes (diagnosis or hemoglobin A_1c_ [HbA_1c_] value of 5.7-6.4) at some point since 2015. Participants will be excluded if they have type 2 diabetes or any other major health condition (eg, cancer or pregnancy) or life situation (eg, incarceration) that could be a confounder or significantly alter the content of Facebook posts.

### Procedures

The study has been approved by the institutional Human Subjects Committee, and the family medicine clinic provided a letter of support.

Participants will be recruited through Idaho State University’s Clinical Research Center at a family medicine clinic. Electronic medical record queries will be utilized to identify potential participants who would qualify for the study. Per protocols of past studies conducted by the Clinical Research Center, potential participants will be mailed a recruitment letter (see Multimedia Appendix 1) providing them with a brief description of the study and a return response card (with instructions that the card be returned by a certain date). The letter will include a statement that the clinic study coordinator will call the potential participant if the card is not returned by the predetermined date indicating that they are not interested. The letter will include an explanation of the call and can be avoided by returning the card (selecting the response that they are not interested in participating) or calling the staff to decline participation. The study coordinator will call potential participants who return the response card indicating interest in the study or who do not return the response card by the requested date. Potential participants who express interest in the study will have the protocol of the study explained to them and will take part in a screening via a telephone call to determine eligibility. This screening (see Multimedia Appendix 2) will verify that participants are eligible for the study. Eligible participants will be scheduled for a clinic visit with the study coordinator.

During the clinic visit, participants will complete formal written informed consent and a set of questionnaires (see Measures below). These questionnaires will be programmed with Qualtrics, and participants will complete the questionnaires over the Web on one of the Clinical Research Center’s computers. Since participants will need to be Facebook users to be eligible for this study, we do not foresee any issues with participants completing Web-based questionnaires; however, paper copies of these questionnaires will be kept ready in case any participant does not want to complete questionnaires over the Web or technical difficulties arise. Participants will be thanked for their participation and provided with a US $25 gift card.

**Figure 1:**
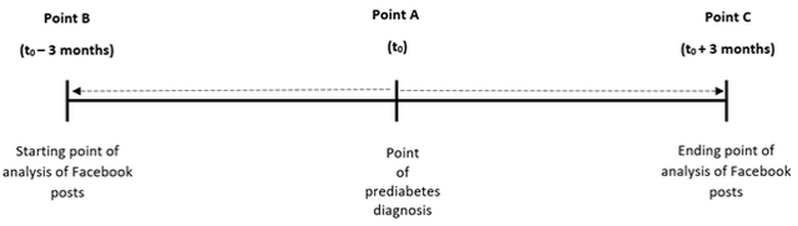
Data collection timeline.

There is an item on the questionnaires that asks participants to provide us with their social media profile name on Facebook. After their clinic visit, we will send each participant a friend request (from a skeletal Facebook account created for this study, with a generic name and no mention of prediabetes, the research center or institution, or other research-related topics). We will then code all Facebook posts made in the 3 months before through the 3 months after their prediabetes diagnosis. Each post will be considered a single data point that will be attributed to the participant. Posts will be coded as they relate to prediabetes predictors. Once all Facebook posts have been coded, we will unfriend the participant and mail them a second US $25 gift card. Figure 1 presents the timeline of data collection.

### Measures

During the clinic visit, participants will complete a set of questionnaires. These include the Facebook Intensity Scale [20], which has been modified for other diabetes social media research [21]; the Diabetes Online Community Engagement Scale [21], modified to be about the Web-based community (as participants will have prediabetes); and the Computer-Mediated Social Support Scale [22]. Minor wording modifications were made to make the questionnaires appropriate for prediabetes (instead of diabetes) and social media. Additionally, we will ask participants about their use of health applications, as past research has shown that these can be helpful in the context of chronic health management [23,24]. Participants will also be asked to provide demographic and other information, including their profile name on Facebook.

For each participant, we will record their total number of Facebook friends, the number of family members on their Facebook profile, their posted relationship status (if any), and the date they joined Facebook or made their first Facebook post (if the joined date is not available). Each Facebook post from the 3 months before through the 3 months after prediabetes diagnosis will also be coded. This coding includes metadata on the post such as the time, date, and type of post (eg, text or video), the viral nature of the post (eg, the number of likes and if the post was shared from or to another page), and the nature of comments made about the post (eg, whether social support was provided and, if so, the type of social support). Additionally, the post’s content will be coded so we can record mentions of symptoms (eg, hunger, fatigue, and negative mood), lifestyle factors (eg, exercise, eating, alcohol, smoking, and self-care), medical experiences (eg, treatment and interaction with health care providers), health tracking (eg, physical activity monitor and glucose), the health of others known to the participant, and additional content (eg, current affairs, religion, and games). Any photos posted by the participant will also be coded on a number of measures including social variables (eg, if the photo was of a group, if the participant was part of the group, or if the participant was tagged) and health variables (eg, if the photo was health-related, if the photo was of food, and the type and composition of the food).

## Results

### Anticipated Timelines

The project was funded for 2015-2018, and enrollment will be completed by the end of 2018. To date, we have enrolled 18 out of the 20 anticipated participants and coded 9 participants’ data.

We anticipate that all clinic visits will be complete by 2018, and all coding of Facebook posts and analyses of the data will be complete by 2019. The first results are expected to be submitted for publication in 2019.

### Planned Data Analyses

A conventional content analysis approach will be used to directly code the Facebook text data [25]. An abstraction of the Facebook postings by several independent researchers will be completed to develop a category scheme to enhance the study reliability [26]. The research team will compare results and will work together to assure high intercoder reliability. This scheme will be used to facilitate the coding of the study data (text, photographs, images, and videos). A comprehensive codebook will be developed that will include the definitions for the categories used in the coding [27]. Major themes will be determined from the data based on a significant proportion of postings identifying the same issue [28]. A thematic approach will be used for the data analysis; the Facebook postings will be read and repeatedly reread for content. Exemplar quotations will be established for each category and theme [25]. Quotes that were common across the participants will be isolated and presented in the findings to show a connection with the data. The findings will be confirmed by sharing with the volunteer participants. Once the results are confirmed, a detailed presentation of the findings in a publishable format will be developed to share the results of the research.

Additionally, a quantitative analysis approach will be used to help characterize the participants and their data. Since this is a pilot study and the first step in attempting to determine an initial taxonomy of prediabetes indicators, we plan on generating a full snapshot of our participants and their Facebook postings by examining all their characteristics over the Web and their responses on self-report items (eg, demographics, means and SDs for scores on all questionnaires, means and SDs for the number of Facebook friends, etc). We will also conduct exploratory paired samples *t* tests to examine potential differences between the 3 months prediagnosis and the 3 months postdiagnosis (eg, the number of posts, tags, comments, and type of post, etc).

## Discussion

### Principal Considerations

This pilot study aims to enroll 20 participants. Data from these participants and their 6 months of Facebook behavior should provide an adequate initial taxonomy of prediabetes indicators. These data will then support future studies with larger samples including testing the utility of the taxonomy on predicting prediabetes status.

### Knowledge Translation

It is anticipated that this study will lead to future research in the field of infodemiology specific to furthering the examination of prediabetes as well as other health issues. Examining social media data may support precision health efforts. Precision medicine is more than “-omics” [29], and examination of big data such as social media postings, may support innovative interventions in which individualized care can be provided through identification of social health behaviors. Perhaps one day, the social behavior that people exhibit over the Web could be predictive of serious health issues, especially diseases that are preventable, such as type 2 diabetes. These identified people could then receive tailored messaging, which has shown to improve outcomes in those with chronic conditions [30] and could be used for primary and secondary prevention. Social media technologies that are commonly used by millions could be crucial in reversing the trends of prediabetes, obesity, and other health concerns in the United States and globally.

While our study attempts the novel approach of taking advantage of behavior that happens in a natural setting (individuals making Facebook posts) to predict prediabetes and, thus, has significant potential to public policy and health, there are also some potential limitations. First, it is possible that the findings may not necessarily generalize to users of all social media (eg, Twitter, Google+, and Instagram). More research would be needed to show that our findings apply across social media platforms. Second, our study is based on respondents in Southeast Idaho, and, thus, it is possible that the findings may not necessarily generalize to individuals elsewhere. Further research would help to test how well our findings hold in other settings. Third, there is also a potential for bias in that there could be a potential for self-selection bias; respondents who are tech-savvy, have high levels of computer self-efficacy, or those with low levels of privacy concern could possibly characterize those that opt to participate in this study. In qualitative research, there is always the potential for researcher bias and certainly in the interpretation of images and words. To control for this potential bias, our research team agreed to use bracketing, or suspense of our personal beliefs and bias, throughout both the data collection and analysis and also in the presentation of our findings. Individually, we also agreed to challenge one another if bias was perceived. To increase objectivity and data consistency, we also determined our coding protocol for the project prior to data collection.

Given the unique nature of our study, where the purpose is to understand whether one’s posting could serve as an index to predict the onset of prediabetes, we think that the design we intend to employ is appropriate. Additional research with a more diverse population of respondents would help assess the validity of the findings.

### Conclusions

The current project aims to develop an initial taxonomy for prediabetes indicators among Facebook users and to help us better understand the social media postings of those with prediabetes. These prediabetes indicators and initial taxonomy are crucial to supporting future larger-scale studies that can advance this programmatic line of research. The ultimate goal of this research will be to develop an automated method to identify social media users who are likely to have prediabetes. This would be especially helpful in the cases of those who have prediabetes but do not know of their health condition, as identification can lead to recommendations (eg, suggesting they be tested) and efforts that can prevent the progression of prediabetes to diabetes.

## Appendix

Multimedia Appendix 1 Deidentified recruitment letter.

Multimedia Appendix 2 Deidentified screening.

## Abbreviations

HbA_1c_: hemoglobin A_1c_
PCP: primary care provider
