# Supplementary material for: Predicting Prediabetes Through Facebook Postings: Protocol for a Mixed-Methods Study
Source: JMIR Res Protoc. 2018 Dec 14;7(12):e10720. doi: 10.2196/10720 (PMC6315248; doi:10.2196/10720)
Supplement: Multimedia Appendix 1 [file resprot_v7i12e10720_app1.pdf]

DATE

Address

Dear XXXXX:

We are writing to invite you to participate in a research study. The purpose of this research study is to learn about the social media use of those who have or recently have had prediabetes. We are inviting you to participate in this research study because you are a patient at XXXXX who meets some of the study's inclusion criteria. Up to 20 patients at XXXXX will take part in this study.

If you agree to take part in this study, we will collect information from your medical record and your past social media postings (this includes your Facebook and other social media screen names). You will also be asked to make 1 visit with the clinic's Study Coordinator. This visit should take about 30 minutes. During this visit we will go over the consent form and you will be asked to provide demographic information and answer questions about your experiences, thoughts, and use of social media. Depending on what information is available/not available from your medical records, you may also be asked to complete a hemoglobin A1C blood test during your visit. This is a simple test that measures blood sugar levels and will involve a finger prick. You can eat and drink normally before the test and you can return to your usual activities immediately afterwards.

You will not have any additional costs for participating in this research project. You will receive a $25 gift card (e.g., Amazon, Visa) at the clinic visit and a second $25 gift card (via mail) at the completion of the study, once all data has been collected. Taking part in this research is voluntary. You may choose not to take part at all. If you choose to participate, you may end your participation at any time and for any reason.

If you are a Facebook user and interested in participating in this study or hearing more information about this study, please contact me at [phone number]. Alternatively, you may mark the "I am interested" box on the provided self-addressed **postage paid** post card and mail it. I will then contact you at the phone number you provide to discuss the study in more detail.

If you do not wish to participate in this study, please mark the "I am not

interested” box on the enclosed post card and return it. If we have not received the enclosed self-addressed post card from you within 10 days, I will make 3 attempts to contact you by phone regarding your potential participation in the study.

Questions are encouraged. If you have any questions about this research project, please contact me at [phone number]. If you have questions about the rights of research subjects please contact [name, address, and phone number of IRB/Human Subjects Committee chair].

Thank you very much for your consideration.

Sincerely,

[Study Coordinator’s information]
