# Supplementary material for: Predicting Prediabetes Through Facebook Postings: Protocol for a Mixed-Methods Study
Source: JMIR Res Protoc. 2018 Dec 14;7(12):e10720. doi: 10.2196/10720 (PMC6315248; doi:10.2196/10720)
Supplement: Multimedia Appendix 2 [file resprot_v7i12e10720_app2.pdf]

Social Media and Prediabetes Phone Screening

Eligible: ☐ No ☐Yes<br>If No, reason:	Screened by:______________________________<br><br>Date screened:_____________________________<br><br>Scheduled session date: ___/___/___ Time: ______

Hello, this is ________from XXXXX. I am contacting you because you are eligible for a research study. We sent you a postcard regarding this study a little while ago [note here if they did not return this card to us and we are following up, or if they did return it to us expressing interest/asking us to contact them]. The purpose of the study is investigate social media usage among those who have or have recently had prediabetes. If you choose to participate in this study we would ask for permission to access some of your medical records and your past social media postings (e.g., on Twitter, Facebook etc.). We would also ask you to come to XXXXX for a brief visit to go through a consent form and to fill out some questionnaires. This should take about 30 minutes. You will be compensated with a $25 gift card at the end of the visit and another $25 gift card upon completion of the study. Is this something that you would be interesting in doing?

☐ Yes ☐ No (INELIGIBLE)

<u>Note: Some items may be filled out by staff based on records prior to this phone screen.</u>

1) What is your age?

☐ Between 18 and 89 ☐ <18 or >89 (INELIGIBLE)

2) Do you currently use Facebook?

☐ Yes ☐ No (INELIGIBLE)

3) Are you proficient in English including able to read English?

☐ Yes ☐ No (INELIGIBLE)

4) Do you currently have type 2 diabetes?

☐ No ☐ Yes (INELIGIBLE)

5) Are you currently receiving emergency medical care and/or terminally ill?

☐ No ☐ Yes (INELIGIBLE)

6) Women only (men, skip to Q6)

a) Are you currently pregnant or nursing?

☐ No ☐ Yes (INELIGIBLE)

7) Do you currently reside in a nursing home or other similar institution?

☐ No ☐ Yes (INELIGIBLE)

8) Are you currently a prisoner, parolee, or incarcerated person?

☐ No ☐ Yes (INELIGIBLE)

9) Do you currently work for [entity conducting research or funding agency]?

☐ No ☐ Yes (INELIGIBLE)

IF ALL ABOVE ANSWERS ARE ELIGIBLE BUT PATIENT ONLY HAS 1 HgA1C ON FILE: Based on your answers you may be eligible for this study, if you are able to come in during one of the following timeslots: [provide timeslots that fall within 8-16 weeks of their 1st HgA1C measure. If the patient cannot make it during this timeframe, they are ineligible. If they can make the timeslot, read the eligible script below and provide information to our location]

IF ALL ABOVE ANSWERS ARE ELIGIBLE AND PATIENT HAS 2 HgA1Cs on file: Congratulations! I am happy to tell you that you are eligible for the study. I'd like to schedule you for your study session. Let me tell you about some available timeslots in the near future, and you can let me know if any of these work for you. [schedule the appointment or find a time to re-contact them to schedule].

The study will take place at XXXXX[Check if they know where this is - provide directions and parking information as needed]

Do you have any other questions? [Answer any questions, refer them to researchers and IRB as needed] Great! We look forward to seeing you [timeslot they signed up for], please let us know if you will need to reschedule. Thanks.

IF INELIGIBLE:

I'm sorry, unfortunately based on some of your responses to these questions you are not eligible to participate in this particular study. Thank you so much for taking the time to answer these questions.
